# A Comparative Assessment of MR BI-RADS 4 Breast Lesions With Kaiser Score and Apparent Diffusion Coefficient Value

**DOI:** 10.3389/fonc.2021.779642

**Published:** 2021-12-02

**Authors:** Lingsong Meng, Xin Zhao, Lin Lu, Qingna Xing, Kaiyu Wang, Yafei Guo, Honglei Shang, Yan Chen, Mengyue Huang, Yongbing Sun, Xiaoan Zhang

**Affiliations:** ^1^ Department of Radiology, The Third Affiliated Hospital of Zhengzhou University, Zhengzhou, China; ^2^ Magnetic Resonance (MR) Research China, General Electric (GE) Healthcare, Beijing, China; ^3^ Department of Magnetic Resonance Imaging (MRI), The First Affiliated Hospital of Zhengzhou University, Zhengzhou, China

**Keywords:** Kaiser score, apparent diffusion coefficient, breast cancer, BI-RADS, specificity

## Abstract

**Objectives:**

To investigate the diagnostic performance of the Kaiser score and apparent diffusion coefficient (ADC) to differentiate Breast Imaging Reporting and Data System (BI-RADS) Category 4 lesions at dynamic contrast-enhanced (DCE) MRI.

**Methods:**

This was a single-institution retrospective study of patients who underwent breast MRI from March 2020 to June 2021. All image data were acquired with a 3-T MRI system. Kaiser score of each lesion was assigned by an experienced breast radiologist. Kaiser score+ was determined by combining ADC and Kaiser score. Receiver operating characteristic (ROC) curve analysis was performed to evaluate the diagnostic performance of Kaiser score+, Kaiser score, and ADC. The area under the curve (AUC) values were calculated and compared by using the Delong test. The differences in sensitivity and specificity between different indicators were determined by the McNemar test.

**Results:**

The study involved 243 women (mean age, 43.1 years; age range, 18–67 years) with 268 MR BI-RADS 4 lesions. Overall diagnostic performance for Kaiser score (AUC, 0.902) was significantly higher than for ADC (AUC, 0.81; *p* = 0.004). There were no significant differences in AUCs between Kaiser score and Kaiser score+ (*p* = 0.134). The Kaiser score was superior to ADC in avoiding unnecessary biopsies (*p* < 0.001). Compared with the Kaiser score alone, the specificity of Kaiser score+ increased by 7.82%, however, at the price of a lower sensitivity.

**Conclusion:**

For MR BI-RADS category 4 breast lesions, the Kaiser score was superior to ADC mapping regarding the potential to avoid unnecessary biopsies. However, the combination of both indicators did not significantly contribute to breast cancer diagnosis of this subgroup.

## Introduction

Worldwide, breast cancer is the most frequently diagnosed malignant tumor in women and is currently the cause of most cancer-related death (1, 2). Dynamic contrast-enhanced (DCE) MRI is an effective tool in distinguishing malignant and benign breast lesions with high sensitivity (3–5). The American College of Radiology (ACR) Breast Imaging Reporting and Data System (BI-RADS) lexicon can provide a standardized and structured description for breast lesions (6). While the BI-RADS category 4 lesions are suspicious of malignancy, they could be recommended for biopsies. The probability of malignancy of BI-RADS 4 lesions varies from 2% to 95% (5, 7), indicating that a large number of benign lesions would receive unnecessary invasive procedures. This will increase the psychological and financial burden for patients. It is necessary to explore a new problem-solving method to improve the diagnostic performance in the assessment of BI-RADS 4 breast lesions.

As a clinical decision rule, Kaiser score (a clinical scoring system) incorporating several BI-RADS diagnostic criteria has demonstrated robust performance in the assessment of breast lesions with excellent sensitivity and specificity (8–13), which could potentially avoid unnecessary biopsies. The Kaiser score consists of 11 rating categories ranging from 1 to 11, with each category corresponding to a distinct likelihood of malignancy (13). If the score exceeds 4, a biopsy is recommended (8, 9).

Diffusion-weighted imaging (DWI) has been widely used for the assessment of breast disease (14–16). The apparent diffusion coefficient (ADC) value derived from DWI data can quantitatively reveal the microstructure changes in biological tissues (16, 17). In general, the ADCs in benign lesions were significantly higher than that of malignant ones (14, 18). Consequently, the findings with high ADCs (greater than a cutoff value) could potentially be regarded as benign lesions, which may avoid unnecessary interventions (15, 19). Baltzer et al. (15) pointed out that ADC >1.4 × 10^−3^ mm^2^/s was considered an effective method for the exclusion of malignancy with a sensitivity of 100%. Clauser et al. (19) found that application of the ADC cutoff value (1.5 × 10^−3^ mm^2^/s) could downgrade the BI-RADS 4 lesions and potentially reduce unnecessary biopsies by 32.6%.

Applying Kaiser score (8, 9, 20) and ADC (19, 21, 22) to reduce unnecessary breast biopsies have been independently validated. Recently, a multicentric study reported that combining these two parameters did not improve diagnostic performance when evaluating breast lesions (10). The study included the breast lesions initially assigned as BI-RADS 0, 4, or 5 at mammography and/or breast ultrasonography. We wonder whether integrating both indicators would improve the diagnostic performance in the assessment of BI-RADS 4 breast lesions on CE-MRI.

Therefore, the purpose of this study was to assess the diagnostic performance of the combination of ADC and Kaiser score for MR BI-RADS 4 breast lesions and to compare it with the diagnostic performance of Kaiser score alone. In addition, the effects of background parenchymal enhancement (BPE) on the performance of the combined indicator were also investigated.

## Materials and Methods

### Study Population

**Figure 1:**
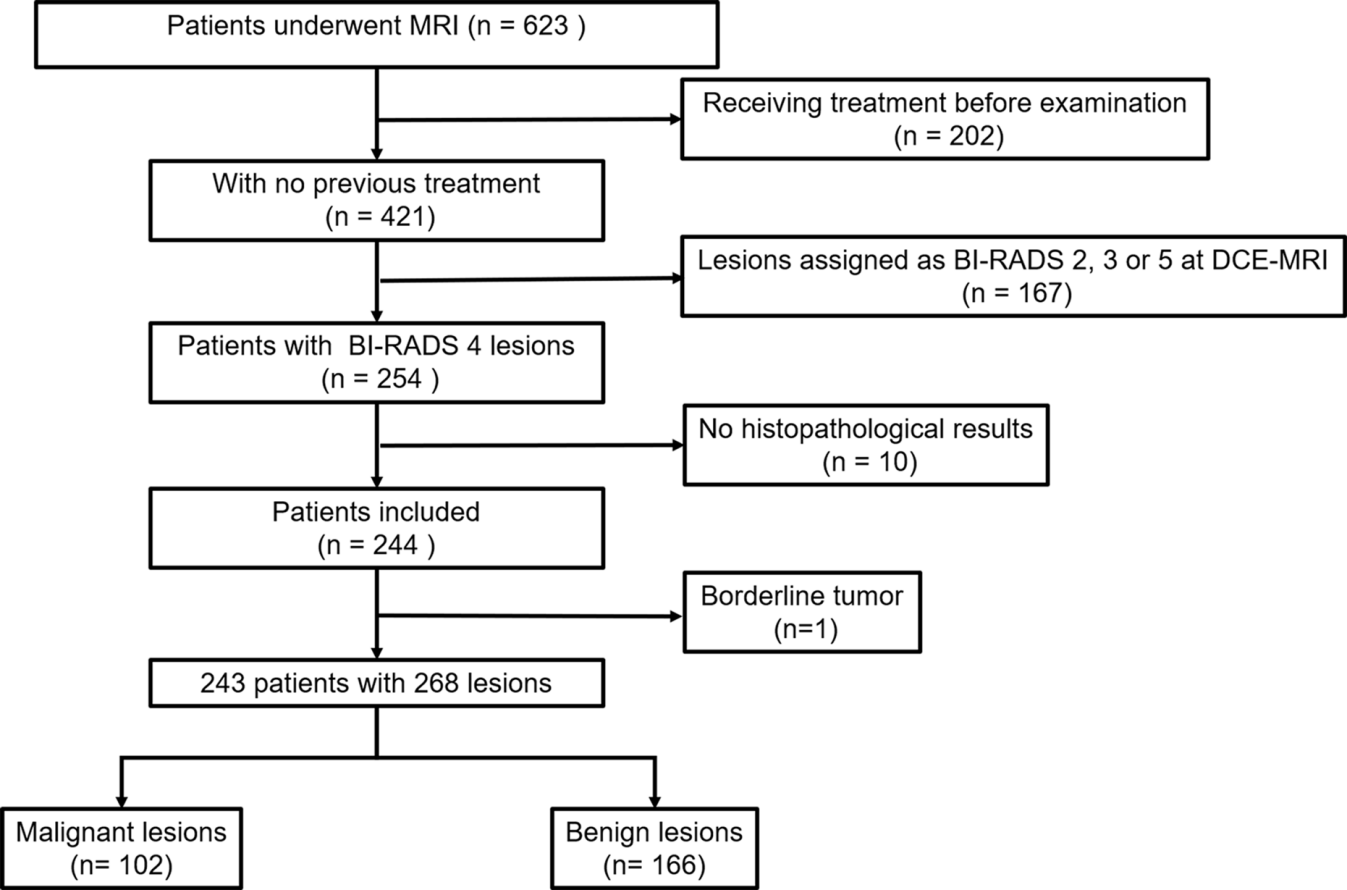
Flowchart shows the data selection in this study.

This retrospective study was approved by our Institutional Review Board, and written informed consent was waived. All patient data were obtained from Picture Archiving and Communication Systems (PACS) and Electronic Medical Record System (EMRS) at our institution. From March 2020 to June 2021, we consecutively reviewed 623 female patients who underwent MRI examinations. Three hundred eighty of these patients were excluded because of the following reasons: (1) receiving chemotherapy or surgery treatment before MR examination (n = 202); (2) lesions assigned as BI-RADS category 2, 3, or 5 at DCE MRI (n = 167); (3) without available histopathological results (n = 10); and (4) borderline tumor (n = 1). Finally, a total of 243 patients with 268 lesions were included in our study (**Figure 1**).

### MRI Protocol

All image data were acquired with a 3-T MRI system (SIGNA Pioneer, GE Healthcare, Waukesha, WI, USA) with an eight-channel breast coil. All patients were examined with the state-of-the-art MRI protocol (3) in the prone position. Detailed parameters are summarized in **Table 1**.

**Table 1:** MRI protocol parameters.

Parameters	T2WI	DWI	DCE
Scan plane	Axial	Axial	Axial
Sequence	FFS	EPI	SPGR
TR/TE, ms	7,281/77	3,000/72	4.7/1.7
Flip angle, °	111	90	15
NEX	2	5	1
AQM	512 × 512	130 × 128	160 × 288
FOV, cm^2^	36 × 36	36 × 36	36 × 36
Slice thickness, mm	5	5	1.2
Slice gap, mm	1	1	0
Number of slices	32	32	134
Fat suppression	ON	ON	ON
b-value, s/mm^2^	–	0/800	–
Acquisition time, min	3:17	2:42	4:33
Contrast agent (only DCE)	Omni-Scan (GE Healthcare, Shanghai, China), 0.1 mmol/kg body weight		

TR, repetition time; TE, echo time; NEX, number of excitations; AQM, acquisition matrix; FOV, field of view; T2WI, T2 weighted imaging; DWI, diffusion-weighted imaging; DCE, dynamic contrast-enhanced; FSE, fast spin echo; EPI, single-shot echo planar imaging; SPGR, spoiled gradient echo.

### Reference Standard

The histopathology of all lesions was regarded as the reference standard. All lesion specimens were obtained by biopsy or surgery, which were subsequently analyzed by two board-certified pathologists (with 5 and 10 years of experience, respectively).

### Image Analysis

**Figure 2:**
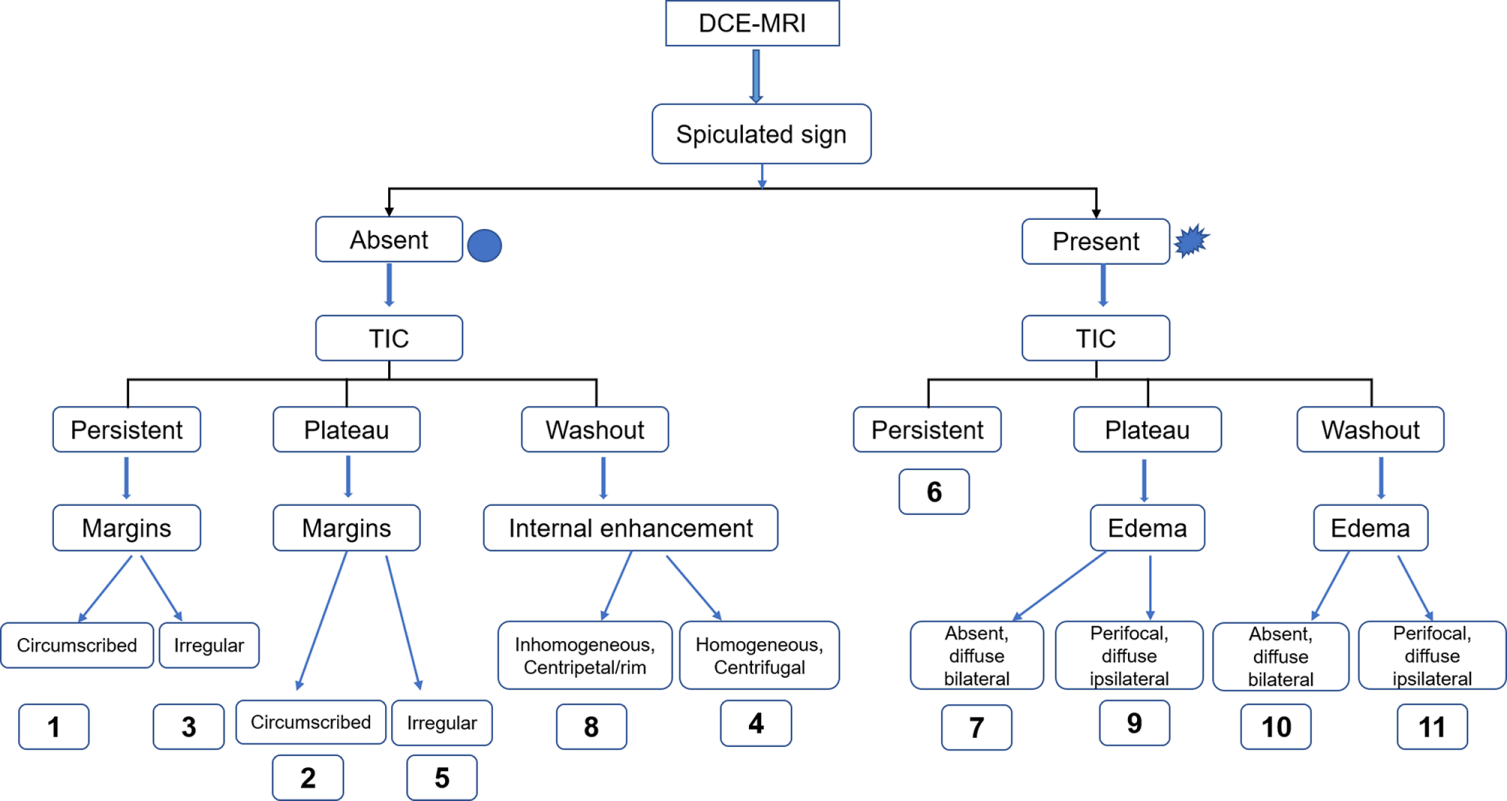
The flowchart of Kaiser score. This flowchart was adapted from a Ref (13). Kaiser score insists of 11 rating categories ranging from 1 to 11, and each of these scores corresponds to a distinct likelihood of malignancy (13). If the score exceeds 4, a biopsy is recommended (8, 9). DCE-MRI, dynamic contrast-enhanced MRI; TIC, time–signal intensity curve.

The BI-RADS categories, BPE, and lesion size of all lesions were extracted from the structured radiology reports. To determine the Kaiser score category, one experienced breast radiologist (QX, with 15 years of experience in reading breast MR images) was required to interpret all examinations according to the Kaiser score system, which consisted of five independent diagnostic criteria [root sign, time–signal intensity curve (TIC) types, lesion margins, internal enhancement patterns, and peritumoral edema], as investigated in previous studies (10, 11, 13, 20, 23, 24). Subsequently, another experienced breast radiologist (LL with 18 years of experience in reading breast MR images) randomly evaluated 100 consecutive cases for assessing interobserver consistency. Both readers were blinded to histopathological characteristics and BI-RADS categorization. The final Kaiser score category of each lesion was calculated and recorded. The flowchart of the Kaiser score system is shown in **Figure 2**.

To measure the ADC of each lesion, all ADC maps were retrieved and transmitted to a dedicated workstation (AW 4.7, GE Healthcare). We used the third method for breast tissue selection as categorized in a meta-analysis by Wielema et al. (25). Two breast radiologists (QX and LL) independently drew regions of interest (ROIs) on the ADC map using the DCE-MRI as references. The ROI included solid areas of the lesion, while the areas with visible necrosis, cystic change, or hemorrhage were excluded. Ultimately, the corresponding ADC was documented. In this study, the average ADCs measured by the two readers were regarded as the final data.

As reported in previous studies (10, 13), the ADC was combined with the Kaiser score to obtain the indicator Kaiser score+. To choose the most effective threshold of ADC for Kaiser score+, we tested four thresholds and found that 1.4 × 10^−3^ mm^2^/s was the optimal one, which was the same as reported in the current literature (10). Then, if the ADC of a lesion exceeded 1.4 × 10^−3^ mm^2^/s, the Kaiser score (threshold >4) was reduced by 1 point (this combination method could give the best diagnostic performance through our test). Otherwise, the Kaiser score stayed the same. The details of the procedure in finding the best combination manner for Kaiser score+ are shown in **Supplementary Material 1**.

### Statistical Analysis

The data were analyzed using SPSS 26.0 (IBM) and MedCalc 19.8 (MedCalc Software). The intraclass correlation coefficient (ICC) (26, 27) was used to analyze interobserver consistency. In this study, the quantitative data that did not conform to normal distribution were expressed in median and interquartile range (IQR) and were compared by using the Mann–Whitney *U* test. Categorical data were analyzed by using the chi-squared test or Fisher’s exact test. The receiver operating characteristic (ROC) curves were plotted to determine the performance of each parameter. Regarding subgroup analysis, the impact of BPE (minimal, mild, moderate, and marked, respectively) on the diagnostic performance of all quantitative parameters was also investigated. The DeLong test was performed to test the differences between independent areas under the ROC curves (AUCs). In this study, the cutoff values (Kaiser score >4; ADC ≤1.4 × 10^−3^ mm^2^/s) were applied (22, 24). Discrimination parameters consisting of sensitivity and specificity were calculated and compared by using the McNemar testing. *p* < 0.05 was considered statistically significant.

## Results

### Patient Demographics

A total of 243 patients (mean age, 43.1 years ± 10.3; age range, 18–67 years) with 268 BI-RADS 4 breast lesions (166 benign and 102 malignant) were included in the study. Among these patients, 25 patients had bilateral breast lesions: 19 had bilateral benign lesions, and 6 had 1 benign lesion and 1 malignant lesion in both breasts. Of the 268 lesions, 102 were malignant (patient age, 48.6 years ± 9.2; age range, 28–67 years), and 166 were benign (patient age, 39.7 years ± 9.4; age range, 18–65 years). The detailed characteristics of patients and lesions are summarized in **Table 2**.

**Table 2:** Characteristics of patients and lesions.

Characteristics	Malignant (n =102)	Benign (n = 166)	*p*-value
Patient characteristic			
Age (years)	48.6 ± 9.2 (28–67)	39.7 ± 9.4 (18–65)	<0.001
Menstrual status			<0.001
Postmenopausal (n = 199)	54 (27.1%)	145 (72.9%)	
Premenopausal (n = 69)	48 (69.6%)	21 (30.4)	
Lesion characteristic			
Root sign			<0.001
Yes (n = 69)	62 (89.9%)	7 (10.1%)	
No (n = 199)	40 (20.1%)	159 (79.9%)	
TIC			<0.001
Persistent (n = 75)	4 (5.3%)	71 (94.7%)	
Plateau (n = 120)	47 (39.2%)	73 (60.8%)	
Washout (n = 73)	51 (69.9%)	22 (30.1%)	
Margins			<0.001
Circumscribed (n = 91)	9 (9.9%)	82 (90.1%)	
Irregular (n = 177)	93 (52.5%)	84 (47.5%)	
Internal enhancement			<0.001
Heterogeneous (n = 213)	99 (46.5%)	114 (53.5%)	
Homogeneous (n = 55)	3 (5.5%)	52 (95.5%)	
Edema			<0.001
Yes (n = 36)	28 (77.8%)	8 (22.2%)	
No (n = 232)	74 (31.9%)	158 (68.1%)	
BPE			0.279
1 (n = 84)	30 (35.7%)	54 (64.3%)	
2 (n = 59)	27 (45.8%)	32 (54.2%)	
3 (n = 73)	30 (41.1%)	43 (58.9%)	
4 (n = 52)	15 (28.8%)	37 (71.2%)	

TIC, time–signal intensity curve; BPE, background parenchyma enhancement.

BPE 1–4 represent minimal (<25%), mild (25%–50%), moderate (50%–75%), and marked (>75%), respectively.

### Interobserver Agreement

The ICC was 0.9126 (95%CI, 0.8702–0.9412) for Kaiser score and 0.9972 (95%CI, 0.9964–0.9978) for ADC. Therefore, the Kaiser score and the ADC measured by the two readers showed excellent agreement.

### Parameter Comparison

In this study, the median ADC of malignant lesions was 0.96 × 10^−3^ mm^2^/s (IQR, 0.84–1.12 × 10^−3^ mm^2^/s), which was significantly lower than that of benign ones with the median ADC of 1.38 × 10^−3^ mm^2^/s (IQR, 1.15–1.62 × 10^−3^ mm^2^/s) (*p* < 0.0001) (**Table 3**). The Kaiser score (median, 8; IQR, 7–9) in cancerous lesions was significantly higher than that in non-cancerous lesions (median, 3; IQR, 2–5) (*p* < 0.0001) (**Table 3**). Regarding Kaiser score+, the value was higher for malignant lesions (median, 8; IQR, 7–9) than for benign lesions (median, 3; IQR, 2–4; *p* < 0.0001) (**Table 3**).

**Table 3:** Comparison of different parameters between benign and malignant lesions.

Parameters	Malignant (n = 102)	Benign (n = 166)	*p*
ADC (×10^−3^ mm^2^/s)	0.96 (0.84–1.12)	1.38 (1.15–1.62)	<0.0001
Kaiser score	8 (7–9)	3 (2–5)	<0.0001
Kaiser score+	8 (7–9)	3 (2–4)	<0.0001

### Comparison of the ROC Curves

**Figure 3:**
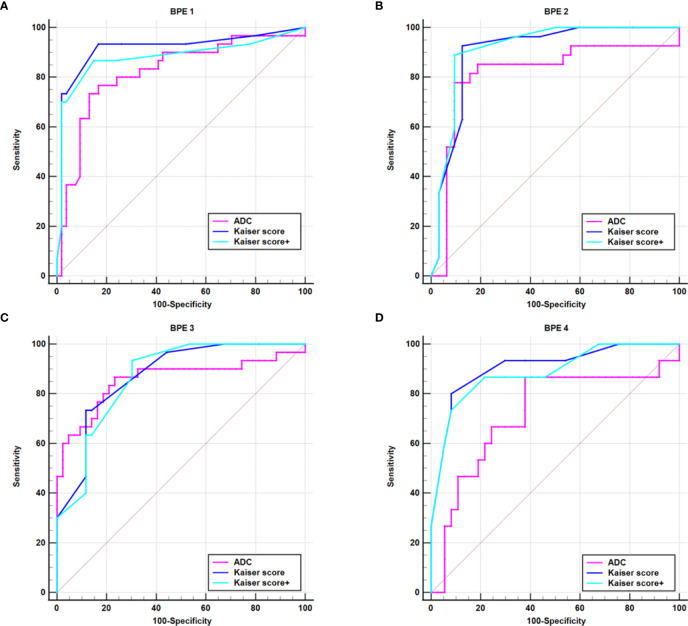
ROC curves show the diagnostic performance of Kaiser score, Kaiser score+, and ADC for different subgroups. **(A)** BPE 1 group, **(B)** BPE 2 group, **(C)** BPE 3 group, **(D)** BPE 4 group. BPE 1–4 represent minimal (<25%), mild (25%–50%), moderate (50%–75%), and marked (<75%), respectively. BPE, background parenchyma enhancement.

For all lesions, the AUCs of Kaiser score+ (AUC, 0.906) and Kaiser score (AUC, 0.902) were higher than that of ADC (AUC, 0.810) (*p* = 0.002, *p* = 0.004, respectively), while there was no significant difference in the AUCs between Kaiser score+ and Kaiser score (*p* = 0.134) (**Table 4**). For the subgroup, such as BPE 4 (marked), the difference in AUCs (Kaiser score+ vs. ADC; Kaiser score vs. ADC) was significant (*p*= 0.004, *p* = 0.007, respectively), while the remaining subgroups were not (all *p* > 0.05). Of note, both Kaiser score+ and Kaiser score showed satisfactory diagnostic performance, regardless of BPE (**Figure 3** and **Table 4**).

**Table 4:** Comparison of ROC curves of Kaiser score, Kaiser score+, and ADC.

		ADC		Kaiser score		Kaiser score+		*p* ^a^	*p* ^b^	*p* ^c^
		AUC (95%CI)	SE	AUC (95%CI)	SE	AUC (95%CI)	SE			
All		0.810 (0.757–0.855)	0.030	0.902 (0.860–0.935)	0.020	0.906 (0.865–0.938)	0.020	**0.004**	**0.002**	0.134
BPE	1	0.822 (0.723–0.897)	0.052	0.895 (0.809–0.952)	0.044	0.893 (0.807–0.950)	0.044	0.243	0.250	0.587
	2	0.816 (0.694–0.905)	0.065	0.903 (0.797–0.965)	0.043	0.912 (0.809–0.970)	0.041	0.219	0.16	0.310
	3	0.857 (0.756–0.928)	0.052	0.873 (0.774–0.939)	0.039	0.885 (0.789–0.948)	0.038	0.808	0.648	0.198
	4	0.719 (0.577–0.835)	0.088	0.908 (0.795–0.970)	0.048	0.909 (0.796–0.971)	0.048	**0.007**	**0.004**	0.900

For all lesions, the AUCs of Kaiser score and Kaiser score+ were higher than that of the ADC (p = 0.004; p = 0.002, respectively). While there were no significant differences between the AUCs of Kaiser score and Kaiser score+ (p = 0.134). BPE 1–4 represent minimal (<25%), mild (25%–50%), moderate (50%–75%), and marked (>75%), respectively.

ADC, apparent diffusion coefficient; AUC, area under the curve; CI, confidence interval; BPE, background parenchyma enhancement.

p^a^ (ADC vs. Kaiser score), p^b^ (ADC vs. Kaiser score+), p^c^ (Kaiser score vs. Kaiser score+).

The bold values indicate statistical significance (p < 0.05).

### Sensitivity and False-Negative Lesions

**Figure 4:**
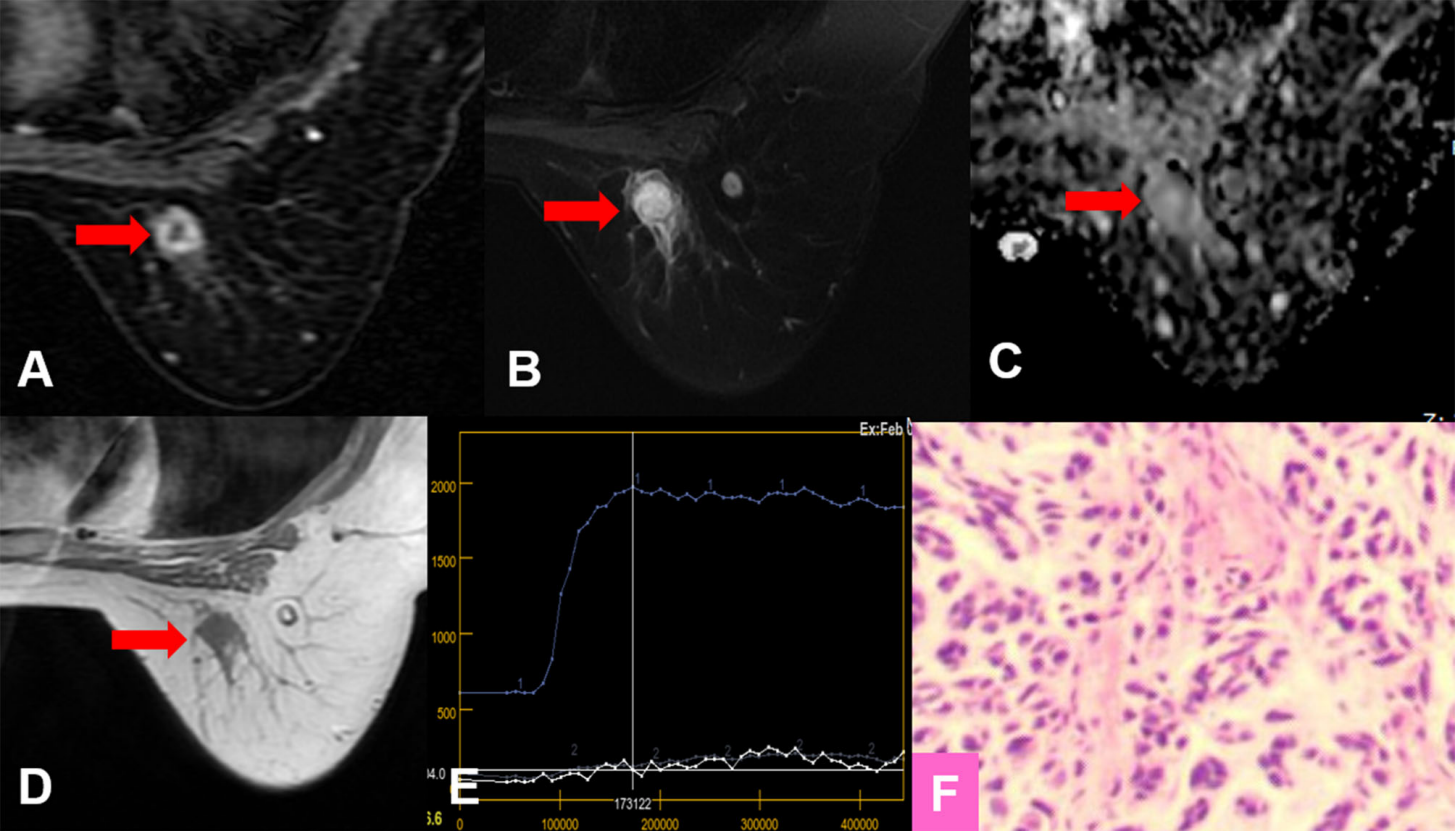
True-positive KS and false-negative KS+ results. A 53-year-old female patient: MRI showed an irregular lesion in the right breast [**(A–D)**, red arrow]. The lesion showed heterogeneous internal enhancement **(A)**, delayed plateau enhancement **(E)**, and without perifocal edema **(B)**. **(A)** Contrast-enhanced MRI, **(B)** T2WI with fat suppression, **(C)** ADC map, **(D)** T1WI, **(E)** TIC curve, **(F)** pathological image (H&E, 100×). Kaiser score = 5, ADC = 1.57 × 10^−3^ mm^2^/s. Histopathology revealed a metaplastic carcinoma **(F)**. Immunohistochemical results are as follows: ER, PR, and HER-2 were negative. Ki-67 expression was high (70%). Vimentin, S-100, and CK-Pan were positive. SMA, Desmin, EMA, CD34, p63, and p40 were negative.

For all lesions, the sensitivities of Kaiser score+ and ADC were lower but not significantly lower than that of Kaiser score (*p* = 1.000, *p* = 0.344, respectively) (**Table 5**). Six breast cancers (three mucinous carcinomas, one ductal carcinoma *in situ*, one invasive ductal cancer, and one medullary carcinoma) were missed using the Kaiser score. Simultaneously, 10 false-negative findings (5 mucinous carcinomas, 3 ductal carcinomas *in situ*, 1 metaplastic carcinoma, and 1 papillary carcinoma) were diagnosed applying the ADC. Of these 16 malignant cases, 3 mucinous carcinomas were missed by both Kaiser score and the ADC. The details of false-negative lesions are provided in **Table 6**. A clinical example is provided in **Figure 4**.

**Table 5:** Sensitivity and specificity for three parameters.

Parameter	Criterion	Sensitivity (TP/TP + FN)	95%CI	Specificity (TN/TN + FP)	95%CI
ADC	≤1.4*	90.2 (92/102)	82.7–95.2	47.6 (79/166)	40.4–56.1
Kaiser score	>4	94.1 (96/102)	87.6–97.8	69.9 (116/166)	62.3–76.7
Kaiser score+	>4	93.1 (95/102)	86.4–97.2	77.7 (129/166)	70.6–83.8

For all lesions, the Kaiser score and Kaiser score+ showed a similar degree of sensitivity (p = 1.000). The diagnostic sensitivity showed no significant difference between Kaiser score and ADC (p = 0.344). Compared with the ADC, the KS acquired a significantly higher specificity (p < 0.0001). Values are given as percentages, absolute numbers in brackets.

TP, true positives; TN, true negatives; FP, false positives; FN, false negatives; CI, confidence interval; ADC, apparent diffusion coefficient.

*Given as ×10^−3^ mm^2^/s.

**Table 6:** Detailed information of the false-negative and false-positive lesions diagnosed by Kaiser score, Kaiser score+, and ADC value.

	False negatives	n	False positives	n
Kaiser score		6		50
	Mucinous carcinoma (G1)	3	Fibroadenoma	10
	Ductal carcinoma *in situ* (G1)	1	Adenosis	15
	Invasive ductal carcinoma (G1)	1	Papilloma	13
	Medullary carcinoma (n.a.)	1	Inflammation	12
Kaiser score+		7		37
	Mucinous carcinoma (G1)	3	Fibroadenoma	5
	Ductal carcinoma *in situ* (G1)	1	Adenosis	10
	Medullary carcinoma (n.a.)	1	Papilloma	10
	Metaplastic carcinoma (n.a.)	1	Inflammation	12
ADC		10		87
	Mucinous carcinomas (G1)	5	Fibroadenoma	26
	Ductal carcinoma *in situ* (G1)	3	Adenosis	28
	Metaplastic carcinoma (n.a.)	1	Papilloma	19
	Papillary carcinoma (n.a.)	1	Inflammation	14

### Specificity and the Potential for Avoiding Unnecessary Biopsies

**Figure 5:**
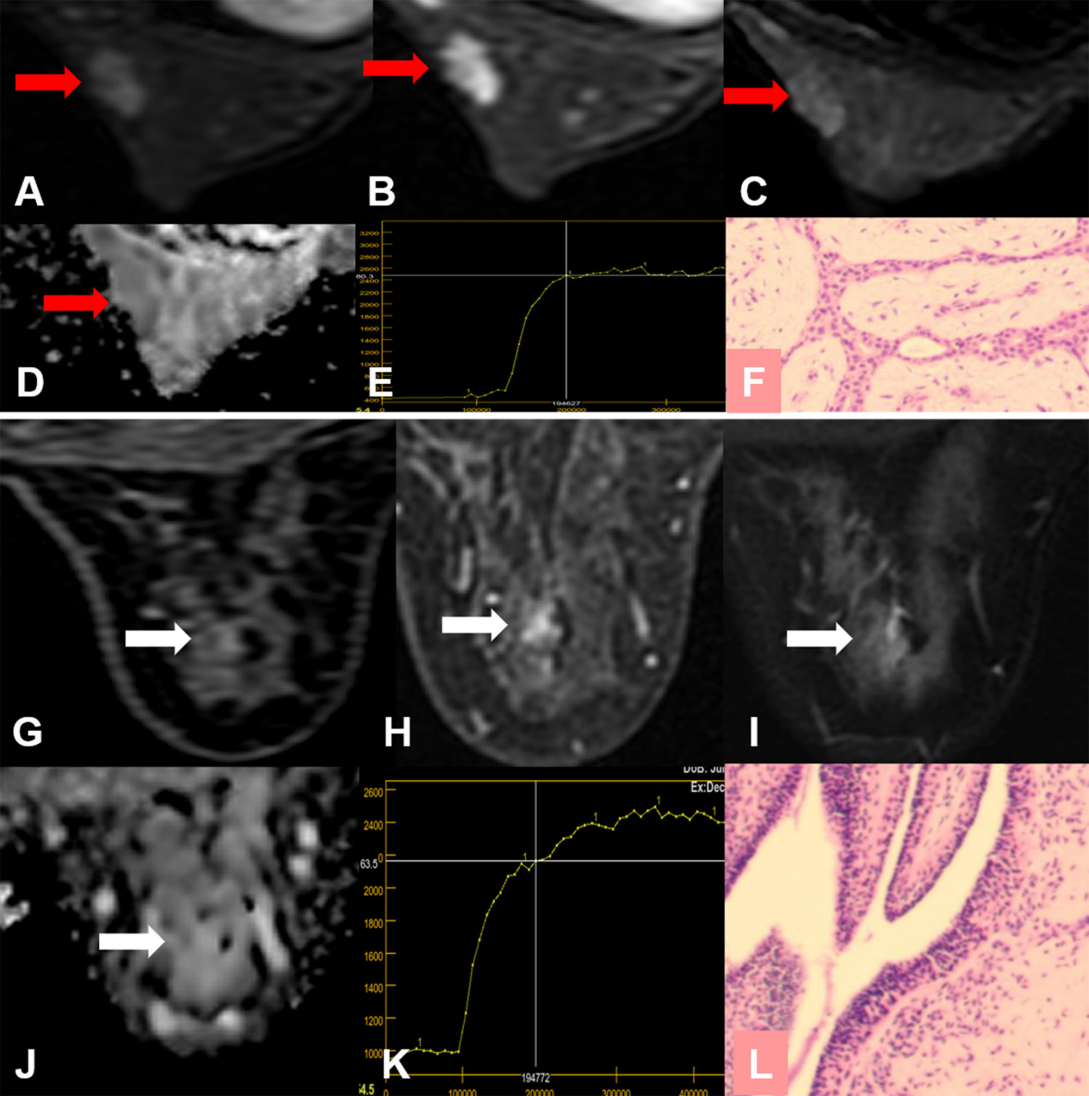
False-positive KS and true-negative KS+ results. **(A–F)** A 24-year-old female patient: MRI showed a mass lesion in the left breast [**(A–D)**, red arrow]. The lesion showed no root sign, a plateau enhancement curve type **(E)**, and irregular margins **(A, B)**. **(A)** Early contrast-enhanced MRI, **(B)** delayed contrast-enhanced MRI, **(C)** T2WI with fat suppression, **(D)** ADC map, **(E)** TIC curve, **(F)** pathological image (H&E, 100×). Kaiser score = 5, ADC = 1.69 × 10^−3^ mm^2^/s. Histopathology revealed a fibroadenoma **(F)**. **(G–L)** A 53-year-old female patient: MRI showed a non-mass lesion in the right breast [**(G–J)**, white arrow]. The lesion showed no root sign, a plateau enhancement curve type **(K)** and irregular margins **(G, H)**. **(G)** Early contrast-enhanced MRI, **(H)** delayed contrast-enhanced MRI, **(I)** T2WI with fat suppression, **(J)** ADC map, **(K)** TIC curve, **(L)** pathological image (H&E, 100×). Kaiser score = 5, ADC = 2.12 × 10^−3^ mm^2^/s. Histopathology revealed adenosis **(L)**.

**Figure 6:**
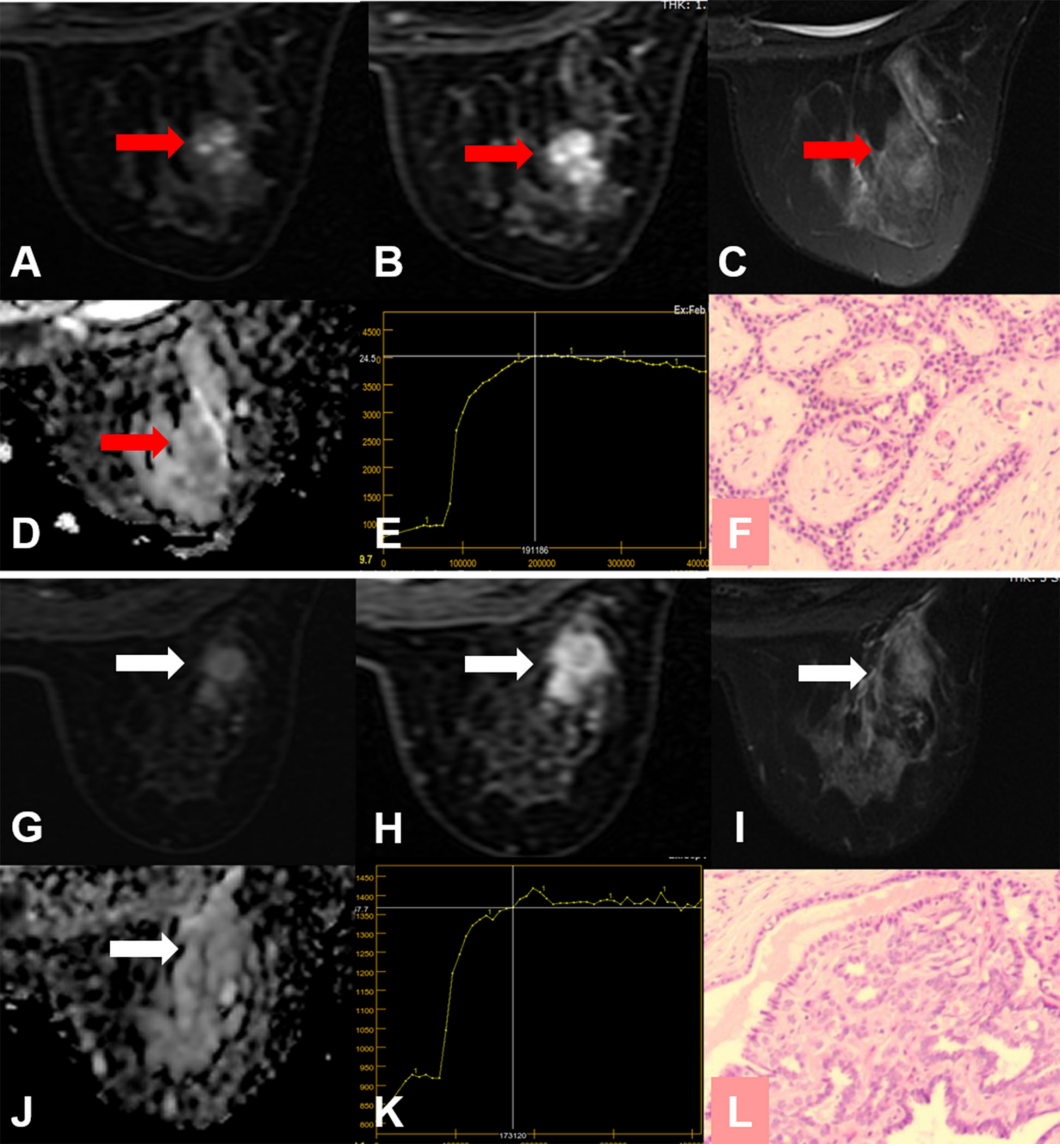
False-positive KS and true-negative KS+ results. **(A–F)** A 24-year-old female patient: MRI showed a mass lesion in the right breast [**(A–D)**, red arrow]. The lesion showed no root sign, a plateau enhancement curve type **(E)**, and irregular margins **(A, B)**. **(A)** Early contrast-enhanced MRI, **(B)** delayed contrast-enhanced MRI, **(C)** T2WI with fat suppression, **(D)** ADC map, **(E)** TIC curve, **(F)** pathological image (H&E, 100×). Kaiser score = 5, ADC = 1.67 × 10^−3^ mm^2^/s. Histopathology revealed a fibroadenoma **(F)**. **(G–L)** A 50-year-old female patient: MRI showed a mass lesion in the right breast [**(G–J)**, white arrow]. The lesion showed no root sign, a plateau enhancement curve type **(K)**, and irregular margins **(G**, **H)**. **(G)** Early contrast-enhanced MRI, **(H)** delayed contrast-enhanced MRI, **(I)** T2WI with fat suppression, **(J)** ADC map, **(K)** TIC curve, **(L)** pathological image (H&E, 100×). Kaiser score = 5, ADC = 1.65 × 10^−3^ mm^2^/s. Histopathology revealed a papilloma **(L)**.

For all lesions, using the ADC could reduce unnecessary biopsies (n = 79/166; 47.6%, 95%CI, 40.4%–56.1%), which was significantly lower than that of Kaiser score (n = 116/166; 69.9%, 95%CI, 62.3%–76.7%) (*p* < 0.0001). The specificity of Kaiser score+ was 77.7% (n = 129/166; 95%CI, 70.6–83.8), which increased by 7.83% compared with Kaiser score alone (*p* = 0.0002) (**Table 5**). Another 13 unnecessary biopsies might be avoided, including adenosis (n = 5), fibroadenoma (n = 5), and papilloma (n = 3). The details of false-positive lesions are provided in **Table 6**. Clinical examples are provided in **Figures 5** and **6**.

## Discussion

In this study, we found that the Kaiser score was superior to ADC mapping regarding the potential to avoid unnecessary biopsies for MR BI-RADS category 4 breast lesions. Potentially, this rate could even be increased by combining ADC value and Kaiser score, however, at the price of a lower sensitivity. The differences between Kaiser score and Kaiser score+ did not show statistical significance.

DWI is a kind of functional imaging technology that has been widely used to improve the diagnostic accuracy of breast MRI (14–16). DWI can quantitatively assess water diffusion in breast tissue by calculating ADC (16, 17). In malignant breast lesions, the signal of DWI increases, and corresponding ADC decreases due to the proliferation of tumor cells, compressed extracellular space, and the hindered diffusion, as shown in this study. As a new diagnostic tool, quantitative ADC is a promising marker in the assessment of breast lesions (28).

Kaiser score is a clinical decision rule that integrates the five most common diagnostic features: root sign, TIC types, lesion margins, internal enhancement patterns, and peritumoral edema (10, 11, 13, 20, 23, 24). We also tested the effectiveness of each feature in the Kaiser score. Further details are shown in **Supplementary Material 2**. Multivariable logistic regression analysis was performed to validate that all of these characteristics except for margins were significantly and independently associated with a breast cancer diagnosis. Moreover, we found that the regression model showed no statistical difference for diagnostic performance in comparison with the Kaiser score. This might explain why the diagnostic performance of the Kaiser score was robust. That might also be the reason that both Kaiser score and Kaiser score+ showed satisfactory diagnostic performance between all BPE subgroups. It is a simple and practical tool for those breast radiologists who need to read images varying in quality. Kaiser score value ranges from 1 to 11, each of which is associated with a distinct probability of malignancy (13). If the score exceeds 4, a biopsy is needed (8, 9). This has been validated in non-mass enhanced lesions on MRI (29), suspicious MRI-only lesions (11), and in lesions that present as mammography-related calcifications (8).

Both ADC and Kaiser score could be regarded as useful imaging biomarkers to benefit clinical decision-making in managing BI-RADS 4 lesions. We analyzed the potential to avoid unnecessary biopsies by using ADC with a threshold of 1.4 × 10^−3^ mm^2^/s and obtained a higher specificity (47.6%) compared with the reports (32.9%) by Dietzel et al. (10). The possible reasons for this result were as follows: first, this study was performed in our single institution, and all data were acquired with one protocol, which may cause the overestimation. Second, we did not analyze the small lesions in this study. When measuring the ADC values of the small lesions, the outlined ROIs may include normal breast tissues, which may lead to higher ADC values, thus the lower performance of ADC. Therefore, excluding such lesions may lead to overestimated results. It is very necessary to be careful about the standardization of ADC. Simultaneously, the above discrepancies may be also related to our higher specificity for Kaiser score+. Our study has done verification of the work but especially focuses on the MR BI-RADS 4 lesions. More critical evaluation will be considered in follow-up work, including studying as many systems, protocols, and centers as we possibly can. Analogous to Dietzel et al. (10), our results showed that the sensitivity with Kaiser score+ also decreased. The missed lesion was a rare finding that exhibited atypical morphological patterns of a malignant lesion. We reviewed the histopathology and found that the lesion was initially diagnosed as carcinosarcoma and finally as metaplastic carcinoma. Metaplastic carcinoma frequently presents with myxoid matrix, intratumoral hemorrhagic changes, and loose edematous stroma (30, 31), which may affect the ADC value. For the same reasons, the morphological features based on DCE-MR may be considered benign. In this study, the missed lesion showed heterogeneous internal enhancement, delayed plateau enhancement, and irregular margins. The Kaiser score was assigned as category 5. The ADC value was 1.57 × 10^−3^mm^2^/s, which was consistent with the reports in the literature (32). Therefore, the Kaiser score+ would lead to the false-negative diagnosis.

Previous studies have confirmed that a high level of BPE was associated with breast cancer (33), and the strong BPE may lead to false-negative or false-positive diagnoses (34–36). However, our results demonstrated that the diagnostic performance of Kaiser score in the assessment of BI-RADS 4 breast lesions did not differ depending on BPE (all *p* > 0.05). Consequently, we speculate that the Kaiser score may provide guidance in cases despite BPE.

Our study also has some limitations. (1) This was a retrospective study conducted at our single institution, and all data were acquired with one protocol, which may lead to the overestimated results. The datasets from multicenters will be prospectively assessed in further research. (2) We did not evaluate the lesions categorized as foci (size <5mm), which might lead to an overestimated performance of the ADC value. The ADCs of foci may be affected by the partial volume effect, and this area warrants further investigation. Previous studies exhibited that the Kaiser score could be applied for assessing foci (9, 10). (3) When measuring the ADC, we outlined the ROI on two-dimensional images and avoided visible necrosis, cystic, or hemorrhagic areas, which might ignore the influence of lesion heterogeneity.

## Conclusion

The Kaiser score is superior to ADC mapping regarding the potential to avoid false-positive biopsies for MR BI-RADS category 4 breast lesions. Potentially, this rate could even be increased by adding ADC measurements in the KS+, however, at the price of lower sensitivity. The combination of both indicators did not significantly contribute to breast cancer diagnosis.

## Data Availability Statement

The raw data supporting the conclusions of this article will be made available by the authors, without undue reservation.

## Ethics Statement

The studies involving human participants were reviewed and approved by the Institutional Review Board of Third Affiliated Hospital of Zhengzhou University (Zhengzhou, China). Written informed consent for participation was not required for this study in accordance with the national legislation and the institutional requirements. Written informed consent was not obtained from the individual(s) for the publication of any potentially identifiable images or data included in this article.

## Author Contributions

LM: conceptualization, software, data curation, formal analysis, data interpretation, manuscript drafting, and manuscript editing. KW, LL, and QX: conceptualization, data curation, data interpretation, manuscript drafting, and manuscript editing. HS, YC, YG, MH, and YS: data curation, data interpretation, analysis, and manuscript editing. XiaZ and XinZ: conceptualization, formal analysis, data curation, data interpretation, and manuscript editing. All authors contributed to the article and approved the submitted version.

## Funding

This research was funded by The National Natural Science Foundation of China. Grant No. 81870983. At the same time, it was also funded by The Department of science and technology of Henan Province. Grant No. 212102310694.

## Conflict of Interest

Author KW was employed by General Electric (GE) Healthcare.

The remaining authors declare that the research was conducted in the absence of any commercial or financial relationships that could be construed as a potential conflict of interest.

## Publisher’s Note

All claims expressed in this article are solely those of the authors and do not necessarily represent those of their affiliated organizations, or those of the publisher, the editors and the reviewers. Any product that may be evaluated in this article, or claim that may be made by its manufacturer, is not guaranteed or endorsed by the publisher.
