# Supplementary material for: A Comparative Assessment of MR BI-RADS 4 Breast Lesions With Kaiser Score and Apparent Diffusion Coefficient Value
Source: Front Oncol. 2021 Dec 2;11:779642. doi: 10.3389/fonc.2021.779642 (PMC8675081; doi:10.3389/fonc.2021.779642)
Supplement: Supplementary file 1 [file DataSheet_1.docx]

**SUPPLEMENTARY MATERIALS 1**

**The Method to Determine ADC Value**

ADC value could be regarded as a useful imaging biomarker to benefit clinical decision-making in managing BI-RADS 4 lesions. In this study, we employed a previously validated ADC (threshold ≤1.4×10^-3^mm^2^/s) for further analysis. Moreover, we also comparatively analyzed the diagnostic efficiency of other reported cut-off values, including 1.3×10^-3^mm^2^/s (1), 1.5×10^-3^mm^2^/s (2), 1.6×10^-3^mm^2^/s (3). The method, however, was used to investigate breast lesions assigned as BI-RADS 0, 4, or 5 based on mammography and/or breast ultrasonography, which was different from ours (BI-RADS 4 based on MRI).

When compared with the use of ADC thresholds (1.5×10^-3^mm^2^/s, 1.6×10^-3^mm^2^/s), the specificity of the threshold of 1.4×10^-3^mm^2^/s increased by 13.86%, 22.29%, respectively. While the sensitivity differences were not significant (P > 0.05) (**Table S1**). Although the application of ADC cut off = 1.3×10^-3^mm^2^/s would increase specificity to 61.45%, which was lower than that of Kaiser score (69.88%). In addition, this came at the cost of 4 additional false-negative lesions (**Table S1**).

**TABLE S1│**Diagnostic Performance of ADC Value for Different Thresholds.

| **Criterion** | **Sensitivity (TP/TP+FN)** | **95% CI** | **Specificity (TN/TN+FP)** | **95% CI** | **+LR** | **95% CI** | **-LR** | **95% CI** |
| --- | --- | --- | --- | --- | --- | --- | --- | --- |
| **≤1.3**^※^ | 86.27 (88/102) | 78.0-92.3 | 61.45 (102/166) | 53.6-68.9 | 2.24 | 1.8-2.8 | 0.22 | 0.1-0.4 |
| **≤1.4**^※^ | 90.20 (92/102) | 82.7-95.2 | 47.59 (79/166) | 40.4-56.1 | 1.74 | 1.5-2.0 | 0.20 | 0.1-0.4 |
| **≤1.5**^※^ | 90.20 (92/102) | 82.7-95.2 | 33.73 (56/166) | 26.6-41.5 | 1.36 | 1.2-1.5 | 0.29 | 0.2-0.5 |
| **≤1.6**^※^ | 91.18 (93/102) | 83.9-95.9 | 25.30 (42/166) | 18.9-32.6 | 1.22 | 1.1-1.4 | 0.35 | 0.2-0.7 |

^※^ Given as ×10^-3^mm^2^/s.

Values are given as percentages, absolute numbers in brackets.

**The Method to Determine Kaiser Score+**

As reported in previous studies(4, 5), the ADC value was combined with the Kaiser score to obtain the indicator Kaiser score+, according to the following rule. If the ADC value of a lesion exceeded 1.4×10^-3^mm^2^/s, the Kaiser score (threshold > 4) was reduced by one point. Otherwise, the Kaiser score stayed the same. This method would obtain a satisfactory Sensitivity (93.14%) and Specificity (77.71%) with the best AUC (0.906) (**Table S2, Table S3**).

**TABLE S2│**Sensitivity and Specificity for Different Indicators.

|  | **Sensitivity (TP/TP+FN)** | **95% CI** | **Specificity (TN/TN+FP)** | **95% CI** | **+LR** | **95% CI** | **-LR** | **95% CI** |
| --- | --- | --- | --- | --- | --- | --- | --- | --- |
| **Kaiser score** | 94.12 (96/102) | 87.6-97.8 | 69.88 (116/166) | 62.3-76.7 | 3.12 | 2.5-4.0 | 0.084 | 0.04-0.2 |
| **Kaiser score minus4** | 88.24 (90/102) | 80.4-93.8 | 78.31 (130/166) | 71.3-84.3 | 4.07 | 3.0-5.5 | 0.15 | 0.09-0.3 |
| **Kaiser score+ （minus1）** | 93.14 (95/102) | 86.4-97.2 | 77.71 (129/166) | 70.6-83.8 | 4.18 | 3.1-5.6 | 0.088 | 0.04-0.2 |
| **Kaiser score minus3** | 91.18 (93/102) | 83.9-95.9 | 77.71 (129/166) | 70.6-83.8 | 4.09 | 3.1-5.5 | 0.11 | 0.06-0.2 |
| **Kaiser score minus2** | 92.16 (94/102) | 85.1-96.6 | 77.71 (129/166) | 70.6-83.8 | 4.13 | 3.1-5.5 | 0.10 | 0.05-0.2 |

Values are given as percentages, absolute numbers in brackets.

**TABLE S3│**AUC for Different Indicators.

| **Variable** | **AUC** | **SE** | **95%CI** |
| --- | --- | --- | --- |
| **Kaiser score** | 0.902 | 0.0197 | 0.860-0.935 |
| **Kaiser score minus4** | 0.894 | 0.0208 | 0.851-0.928 |
| **Kaiser score minus3** | 0.900 | 0.0200 | 0.858-0.933 |
| **Kaiser score minus2** | 0.904 | 0.0197 | 0.863-0.937 |
| **Kaiser score+  (minus1)** | 0.906 | 0.0196 | 0.865-0.938 |

**REFERENCES**

1. Baltzer P, Mann RM, Iima M, Sigmund EE, Clauser P, Gilbert FJ, et al. Diffusion-weighted imaging of the breast-a consensus and mission statement from the EUSOBI International Breast Diffusion-Weighted Imaging working group. *Eur Radiol* (2020) 30(3):1436-50. Epub 2019/12/02. doi: 10.1007/s00330-019-06510-3.

2. Clauser P, Krug B, Bickel H, Dietzel M, Pinker K, Neuhaus VF, et al. Diffusion-weighted Imaging Allows for Downgrading MR BI-RADS 4 Lesions in Contrast-enhanced MRI of the Breast to Avoid Unnecessary Biopsy. *Clin Cancer Res* (2021) 27(7):1941-8. Epub 2021/01/16. doi: 10.1158/1078-0432.CCR-20-3037.

3. Rahbar H, Zhang Z, Chenevert TL, Romanoff J, Kitsch AE, Hanna LG, et al. Utility of Diffusion-weighted Imaging to Decrease Unnecessary Biopsies Prompted by Breast MRI: A Trial of the ECOG-ACRIN Cancer Research Group (A6702). *Clin Cancer Res* (2019) 25(6):1756-65. Epub 2019/01/17. doi: 10.1158/1078-0432.CCR-18-2967.

4. Dietzel M, Krug B, Clauser P, Burke C, Hellmich M, Maintz D, et al. A Multicentric Comparison of Apparent Diffusion Coefficient Mapping and the Kaiser Score in the Assessment of Breast Lesions. *Invest Radiol* (2021) 56(5):274-82. Epub 2020/10/31. doi: 10.1097/RLI.0000000000000739.

5. Dietzel M, Baltzer PAT. How to use the Kaiser score as a clinical decision rule for diagnosis in multiparametric breast MRI: a pictorial essay. *Insights Imaging* (2018) 9(3):325-35. Epub 2018/04/05. doi: 10.1007/s13244-018-0611-8.
