# Supplementary material for: A Comparative Assessment of MR BI-RADS 4 Breast Lesions With Kaiser Score and Apparent Diffusion Coefficient Value
Source: Front Oncol. 2021 Dec 2;11:779642. doi: 10.3389/fonc.2021.779642 (PMC8675081; doi:10.3389/fonc.2021.779642)
Supplement: Supplementary file 2 [file DataSheet_2.docx]

**SUPPLEMENTARY MATERIALS 2**

**The Diagnostic Performance of Kaiser Score was Robust**

In this study, four descriptor groups were significantly and independently associated with a breast cancer diagnosis (**Table S1**). The characteristics with the highest odds ratios (OR) for malignancy were root sign and wash out (OR = 23.132, 35.395, respectively). The margins were not statistically significant (*P* > 0.05). Multivariable logistic regression analysis didn’t identify the statistical difference in diagnostic accuracy compared with the application of the Kaiser score (*P* = 0.067) (**Table S2**). This might explain why the diagnostic performance of the Kaiser score was robust and independent from background parenchymal enhancement (BPE).

**TABLE S1**│Multivariable Logistic Regression Model showed Odds ratios (OR) for Kaiser Score Descriptors with 95% Confidence Intervals and *P*-values.

| Variables | Exp(B) | 95%CI | | *P* |
| --- | --- | --- | --- | --- |
|  |  | *Lower* | *Upper* |  |
| Root sign |  |  |  |  |
| No* |  |  |  |  |
| Yes | 23.132 | 8.637 | 61.953 | <0.0001 |
| TIC |  |  |  |  |
| Persistent* |  |  |  |  |
| Plateau | 6.776 | 1.916 | 23.965 | 0.003 |
| Washout | 35.395 | 9.131 | 137.2 | <0.0001 |
| Internal enhancement |  |  |  |  |
| Homogeneous* |  |  |  |  |
| Heterogeneous | 13.133 | 3.13 | 55.1 | 0.0004 |
| Edema |  |  |  |  |
| No* |  |  |  |  |
| Yes | 3.043 | 1.026 | 9.03 | 0.0449 |

* Reference value.

*CI,* confidence interval; *TIC,* time intensity curve.

**TABLE S2**│ROC Analysis of the Diagnostic Performance for Different Methods.

| Methods | AUC | SE | 95%CI |
| --- | --- | --- | --- |
| Kaiser score | 0.902 | 0.0197 | 0.860-0.935 |
| Model | 0.925 | 0.0177 | 0.887-0.954 |

*AUC,* area under the curve; *SE,* standard error; *CI,* confidence interval; *TIC,* time intensity curve.

*Model* = Multivariable logistic regression model.

The differences in AUCs (Kaiser score vs. model) were not significant (*P* = 0.067).
